# Risk factors affect accurate prognosis in ASXL1-mutated acute myeloid leukemia

**DOI:** 10.1186/s12935-021-02233-y

**Published:** 2021-10-09

**Authors:** Yi Fan, Linxiao Liao, Yajun Liu, Zhenzhen Wu, Chong Wang, Zhongxing Jiang, Shujuan Wang, Yanfang Liu

**Affiliations:** 1 Department of Hematology, The First Affiliated Hospital of Zhengzhou University, Zhengzhou, China; 2 Department of Intensive Care Unit, Zhongshan People’s Hospital, Zhongshan, China; 3 Department of Orthopaedics, Brown University, Warren Alpert Medical School/Rhode Island Hospital, Providence, RI USA

**Keywords:** Acute myeloid leukemia, *ASXL1* mutations, Prognosis, Allogenic hematopoietic stem cell transplantation

## Abstract

**Background:**

The epigenetic regulator additional sex combs-like 1 (*ASXL1*) is an adverse prognostic factor in acute myeloid leukemia (AML). However, the mutational spectrum and prognostic factors of *ASXL1*-mutated (*ASXL1*+) AML are largely unknown. We aim to evaluate the risk factors influencing the prognosis of *ASXL1*+ AML.

**Methods:**

We performed next-generation sequencing (NGS) in 1047 cases of de novo AML and discovered 91 *ASXL1*+ AML (8.7%). The *Log-Rank* test and *Kaplan-Meier* were used to evaluate survival rate, and the *Cox* regression model was used to analyze multivariate analysis.

**Results:**

In a total of 91 *ASXL1*+ AML, 86% had one or more co-mutations. The factors that had adverse impact on overall survival (OS) and event-free survival (EFS) are defined as high risk factors, including age ≥ 60 years, WBC count ≥ 50 × 10^9^/L, *FLT3-ITD* mutations, *RUNX1* mutations, and absence of *AML1-ETO* fusion gene. *ASXL1* mutations without any risk factor were classified as single-hit *ASXL1*+ AML; *ASXL1* mutations accompanied with one of the risk factors was referred to as double-hit *ASXL1*+ AML; *ASXL1* mutations with two or more of the risk factors were designated as triple-hit *ASXL1*+ AML. The combination of these risk factors had a negative influence on the prognosis of *ASXL1*+ AML. The median OS was not attained in single-hit *ASXL1*+ AML, 29.53 months in double-hit *ASXL1*+ AML, and 6.67 months in triple-hit *ASXL1*+ AML (*P* = 0.003). The median EFS was not attained in single-hit *ASXL1*+ AML, 29.53 months in double-hit *ASXL1*+ AML, and 5.47 months in triple-hit *ASXL1*+ AML (*P *= 0.002). Allogenic hematopoietic stem cell transplantation (allo-HSCT) improved the prognosis of double/triple-hit *ASXL1*+ AML patients.

**Conclusions:**

Our study provided new insights into the mutational spectrum and prognostic factors of *ASXL1*+ AML patients. Our primary data suggest that the risk factors in *ASXL1*+ AML contribute to the poor outcome of these patients. The management of *ASXL1*+ AML patients should be based on the risk factors and allo-HSCT is highly recommended for consolidation.

**Supplementary Information:**

The online version contains supplementary material available at 10.1186/s12935-021-02233-y.

## Background

Acute myeloid leukemia (AML) is a group of hematological malignancies with high heterogeneity [1, 2]. Advances in individualized induction regimens with targeted agents and hematopoietic stem cell transplantation (HSCT) for consolidation have significantly improved the results of AML patients. However, the prognosis in some AML cases remains unsatisfactory. Recurrent chromosomal abnormalities and gene mutations have been implicated in leukemogenesis and are employed in the clinic for risk-adopted AML therapy [3]. The favorable risk factors are t(8;21), inv(16)/t(16;16), t(15;17), and *CEBPA* double mutations and *NPM1* mutations, but the adverse risk factors are t(9;22) and mutations in *FLT3-ITD*, *RUNX1*, and *AXSL1 *[4, 5]. It has been established that not all molecular alterations have prognostic and therapeutic implications in AML. The mutations of *CEBPA* showed a favorable prognostic impact on AML only when the mutations occurred at both alleles [6]. The *FLT3-ITD* mutations had a negative prognostic impact when the ratio of the mutant alleles to wild alleles was more than 0.5 [7, 8]. The beneficial prognostic effects of t(8;21) and inv(16)/t(16;16) can be reversed by co-occurring with c-*KIT* mutations [9], and the same goes for *NPM1* co-occurring with *FLT3-ITD* mutations [10]. Agents that target mutations, such as midostaurin on *FLT3-ITD*, can rescue patients from unfavorable outcomes [11, 12]. Based on the understanding of gene mutations in the prognosis of AML, hematologists have used innovative and targeted agents in chemotherapy to improve the outcome of these patients [13]. However, some patients may have multiple gene mutations or risk factors simultaneously. The interaction between mutated genes and other risk factors may affect the prognosis of AML patients. For instance, coexistence of *ASXL1* and *SRSF2* mutations may increase the risk of death in AML patients [14]. Therefore, it is crucial to make precise risk stratification to guide the managements of AML patients. Further research is needed to determine the interaction of co-mutated genes and clinical risk factors in patients carrying certain mutations, such as *AXSL1*, on the prognosis and treatment options.

ASXL1 is the human homologue of the Drosophila Additional sex combs (Asx) [2]. The ASXL family consists of three members (ASXL1, ASXL2, and ASXL3) with conserved domain structures consisting of ASXN, ASXH, ASXM1, ASXM2, and a PHD finger [15]. *ASXL1* encodes a chromatin binding protein of the polycomb group and trithorax complex family [16, 17], which may be involved in epigenetic regulation. *ASXL1* is located on chromosome 20q11. ASXL1 acts as a coactivator for the retinoid receptors including retinoic acid receptor (RAR) and retinoid X receptor through binding with steroid receptor coactivator-1 [18]. Moreover, ASXL1 also cooperates with heterochromatin protein-1 and histone H3 demethylase LSD1 to regulate histone methylation and repress retinoic acid-receptor activity [19]. Germline mutations of *ASXL1* and *ASXL3* can be seen in individuals with congenital abnormalities, such as Bainbridge–Ropers syndrome, while somatic truncation mutants of all three *ASXL* family members are found in human cancer [15]. *ASXL1* is frequently mutated in patients with different types of myeloid malignancies, including myelodysplastic syndromes (MDS), myeloproliferative neoplasms, chronic myelomonocytic leukemia, and AML with MDS-related alterations [20]. *ASXL1* mutations are commonly associated with aggressive behaviors and a poor clinical prognosis across the spectrum of malignant myeloid diseases [21]. In mouse model experiments, *ASXL1* silencing together with oncogenic *NRasG12D* generates hepatosplenomegaly and progressive anemia, emphasizing *ASXL1’s* function in myeloid malignancies [2]. Although the adverse prognostic value of *ASXL1* mutations in AML is obvious, previous studies often focused on comparing the difference between mutated and wild-type *ASXL1* patients. However, the impact of other factors such as variant allele frequency (VAF) and companion gene mutations (co-mutations) on the prognosis of *ASXL1*+ AML needs to be evaluated.

In this study, we comprehensively investigated the mutational spectrum and prognostic factors of *ASXL1+* AML. We also analyzed the interaction of molecular profiles of gene mutation and clinical risk factors on the survival of *ASXL1+* AML patients. Our data demonstrated that the addition of risk factors to *ASXL1* mutations were associated with the adverse outcome of AML patients. Meanwhile, Allo-HSCT and *AML/ETO* fusion gene improved the survival of *ASXL1*+ AML patients. In conclusion, our data provide new evidence for precise risk stratification and optimal treatments of *ASXL1*+ AML.

## Subjects and methods

### Patients

Between May 2016 and January 2020, 1047 cases of de novo AML were examined with next-generation sequencing (NGS) at the First Affiliated Hospital of Zhengzhou University. A total of 91 cases with *ASXL1*+ AML were identified and included in the research. The WHO 2016 edition of classification of myeloid neoplasms and acute leukemia [22] was used to make the diagnosis and classification of AML. According to the 2017 ELN guideline for adult acute myeloid leukemia [4], patients were categorized into three risk groups: favorable-risk, intermediate-risk, and adverse-risk. This study was approved by the Ethics Committee of the First Affiliated Hospital of Zhengzhou University. Following the Declaration of Helsinki, all patients or their legal guardians gave their informed permission.

### Treatment protocols

All-trans retinoic acid and arsenic trioxide-based chemotherapy was used for induction and consolidation therapy in individuals with acute promyelocytic leukemia (APL). Induction chemotherapy regimens for non-APL patients included the DA, IA, and MA regimens, which consisted of a standard dose of cytarabine (Ara-C; 100 mg/m^2^/day for 7 days) combined with daunorubicin (60 mg/m^2^/day for 3 days) or idarubicin (12 mg/m^2^/day for 3 days) or mitoxantrone (10 mg/m^2^/day for 3 days). Patients were given cytarabine (2–3 g/m^2^, once every 12 h for 3 days)-based chemotherapy after remission. The chemotherapy consolidation for older patients was chosen on an individual basis by the specialists. As part of the consolidation process, 12 patients received allo-HSCT. The actual therapy was chosen based on both the doctor’s suggestion and the patient’s desire. The final follow-up for surviving patients occurred in January 2021.

### Fusion genes detection

Fresh bone marrow samples were collected using an Ethylene Diamine Tetraacetic Acid (EDTA) anticoagulant tube. Mononuclear cells were extracted by density gradient centrifugation. RNA was extracted using the standard TRIzol technique (Invitrogen, Carlsbad, CA, USA) and reverse-transcribed into cDNA using a High Capacity cDNA Reverse Transcription Kit (Applied Biosystems, Foster City, CA, USA). Fusion genes were detected by real-time quantitative PCR using Multiplex RT-PCR Fusion Gene Kits (Rightongene, Shanghai, China). A panel of forty-three fusion genes was screened, including *MLL-(AF4, AF6, AF9, AF10, AF17, AF1q, AF1p, AFX, ELL, SEPT6, ENL), NUP98-(HoxA11, HoxA13, PMX1), (NPM, F1P1L1, PML, PRKAR1A, STAT5b, NUMA1, PLZF)-RARα, (ETV6, FIPIL1)-PDGFRA, AML1-(ETO, MTG16, MDS1/EV11), TEL-(JAK2, AML1, ABL), NPM-(ALK, MLF), (DEK, SET)-CAN, SIL-TAL1, E2A-HLF, TEL-PDGFRB, TLS-ERG, CBFβ-MYH11, BCR-ABL, E2A-PBX1.*

### Next-generation sequencing

The mutational hotspots or whole coding regions of 22 genes were assessed by next-generation sequencing, including *FLT3, NPM1, KIT, CEBPA, DNMT3A, IDH1, IDH2, TET2, EZH2, RUNX1, ASXL1, PHF6, TP53, SF3B1, SRSF2, U2AF1, ZRSR2, NRAS, CBL, SETBP1, ETV6*, and *JAK2*. The detection was performed utilizing a Rightongene AML/MDS/MPN Sequencing Panel (Rightongene, Shanghai, China) on an Illumina MiSeq System (Illumina, San Diego, CA) high-throughput sequencing platform. The original data after sequencing was analyzed by bioinformatics using NCBI, CCDS, dbSNP (v138), COSMIC, human genome database (HG19) and other databases to determine the pathogenic mutation site. The average depth of the sequencing was 4837.978Kb, detection sensitivity was ~ 5%. Details on variant calling, filtering, and annotation are detailed in our recently published reports [23].

### Statistical analysis

SPSS software version 26.0 (Chicago, IL, USA) and GraphPad Prism^TM^ 8.01(San Diego, California, USA) were used for the analysis. Continuous variables were presented as mean values ± standard deviation, or median (range) considering whether the data fit a normal distribution or not; categoric measures were summarized with frequency counts and percentages. Overall survival (OS) is defined as the time from diagnosis to death or the time of the last follow-up. Event-free survival (EFS) is defined as the time from diagnosis to relapse, death, or the time of the last follow-up. The *Kaplan-Meier* method was used for survival analysis, and the *Log-rank* test was utilized to assess differences between groups. Univariate analysis and multivariate analysis were performed using the *Cox* proportional hazard regression model. Multivariable analysis including variables with *P*<0.05 in univariate analysis were performed for OS and EFS. A two-sided *P *< 0.05 was regarded as statistically significant.

## Results

### Clinical features of *ASXL1*+ AML patients

*ASXL1* mutations were found in 8.7% (91 of 1047) of the patients in the whole cohort. The median age of the patients was 50 (33–58) years, with 20 cases older than 60 years and 49 cases being male, as indicated in Table 1. The median white blood cell (WBC) count was 7.5 (2.4-33.3) × 10^9^/L, with 18 cases (19.78%) having a value of ≥ 50 × 10^9^/L. Bone marrow blast percentage of more than 80% was seen in 15 cases (16%). According to the 2017 ELN risk criteria, 27 cases (20%) were favorable-risk AML (including 4 cases of APL), 1 case (1%) was intermediate-risk AML, and 63 cases (69%) were adverse-risk AML. Allo-HSCT was applied in 12 patients (13%). Three cases died within 30 days after induction therapy, and 50 cases (63%) died at the end of the follow-up.

**Table 1:** Clinical characteristics of *ASXL1*+ AML

Characteristics	Median (interquartile range) or N (%)
Gender male (n [%])	49 (54%)
Age (years)	50 (33–58)
Age ≥ 60 years (n [%])	20 (22%)
Type (APL vs. non-APL)	4 (4%)
*ASXL1* type (n [%])	
G652S	38 (41.76%)
G642fs	11 (12.09%)
H630fs	8 (8.79%)
* ASXL1* VAF(%)	49.17 (22.9–57.11)
* ASXL1* VAF (≥49.17%)	46 (51%)
WBC counts (× 10^9^/L)	7.5 (2.4–33.3)
WBC counts (≥50 × 10^9^/L)	18 (19.78%)
HGB counts (g/L)	79 (66–93)
HGB counts (≥110 g/L)	13 (14%)
PLT counts ( × 10^9^/L)	48 (20–93)
PLT counts (≥100 × 10^9^/L)	19 (21%)
Bone marrow blasts (%)	51 (26–72)
Bone marrow blasts (≥80%)	15 (16%)
* AML1-ETO*	17 (19%)
* CBFβ-MYH11*	3 (3%)
Risk group	
Favorable	27 (30%)
Intermediate	1 (1%)
Adverse	63 (69%)

*APL* acute promyelocytic leukemia, *VAF* variant allele frequency, *WBC* white blood cell, *HGB* hemoglobin, *PLT* platelet

The molecular mutations of *ASXL1* were detected in 30 different nucleotide sites, all of which were located in exon 12, including G652S (41.76%), G642fs (12.09%), H630fs (8.79%), S1231F and R693X (5.49%), N986S (4.40%), T1139K (3.33%), G643fs and Y591X (2.20%). The distribution of all nucleotide sites was shown in Additional file 1. Most of the patients carried a single-point mutation, 7 (7.69%) patients carried two-point mutations, and one patient carried three-point mutations (G642fs, G643fs and G645fs). The median VAF value of *ASXL1* mutation was 49.17% (1.02–79.28%).

### Companion gene mutations and fusion genes in *ASXL1*+ AML patients

**Fig. 1:**
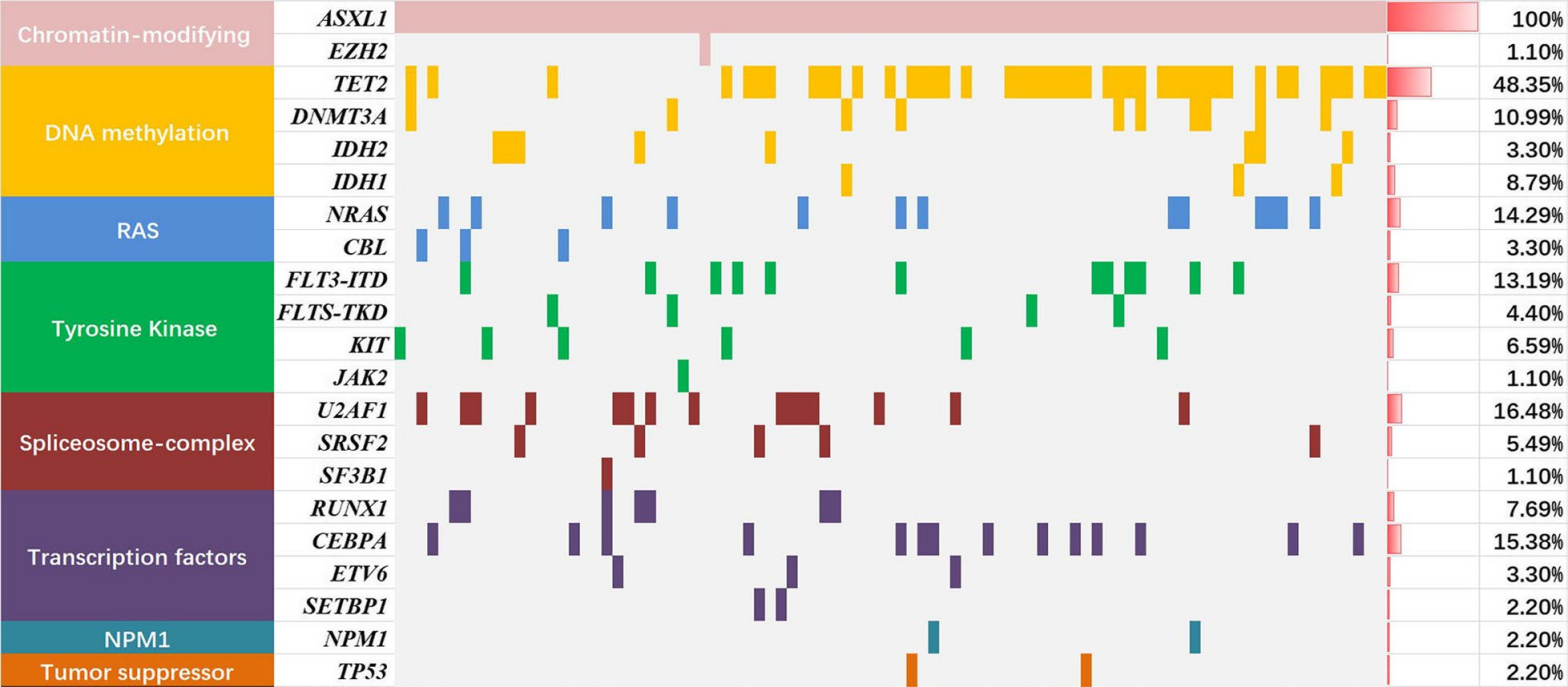
The mutational landscape of 91 *ASXL1*+ AML patients. The landscape displayed all genetic anomalies for each subject. A single patient instance was represented by the boxes in one column. Mutations were color coded according to mutation type. The frequency distribution of all aberrations was depicted by the histogram on the right

One or more co-mutation of genes was observed in 83 patients (86.46%) of *ASXL1+* AML (Fig. 1). *TET2* had the highest mutation frequency (48.35%), followed by *U2AF1* (16.48%), *CEBPA* (15.38%), *NRAS* (14.29%), *FLT3-ITD* (13.19%), *DNMT3A* (10.99%), *IDH2* (8.79%), *RUNX1* (7.69%), *KIT* (6.59%), and *SRSF2* (5.49%). Other mutant genes (including *FLT3-TKD*, *ETV6*, *IDH1*, *CBL*, *SETBP1*, *NPM1*, *TP53*, *EZH2*, *SF3B1*, *JAK*) are found in fewer than 5% of *ASXL1*+ AML patients; *PHF6* and *ZRSR2* mutations are not seen in *ASXL1*+ AML patients.

The fusion genes were screened in 83 of 91 *ASXL1*+AML cases. There were 31 cases (37.35%) with fusion gene mutations, including *AML1-ETO* in 17 cases (20.48%), *PML-RARα* in 4 cases (4.82%), *BCR-ABL*, *MLL-AF9* and *CBFβ-MYH11* in 3 cases (3.61%), *MLL-ELL* in 1 case (1.20%). The remaining 52 cases (62.65%) were with negative fusion genes.

### Risk factors on the prognosis of *ASXL1*+ AML

In order to understand the prognostic impacts of clinical features and molecular profiles on the outcomes of *ASXL1*+ AML patients, we analyzed the risk factors on OS and EFS including gender (female vs. male), age (≥ 60 vs. < 60 years), *ASXL1* nucleotide sites, *ASXL1* VAF (≥ 49.17% vs. < 49.17%), WBC counts (≥ 50 vs. < 50 × 10^9^/L), HGB (≥ 110 vs. < 110 g/L), PLT counts (≥ 100 vs. < 100 × 10^9^/L), bone marrow blasts (≥ 80% vs. < 80%), peripheral blood blasts (≥ 20% vs. < 20%), allo-HSCT (yes vs. no), risk stratification (adverse vs. inter/favorable -risk), *AML1-ETO* fusion gene (positive vs. negative), *CBFβ-MYH11* fusion gene (positive vs. negative), and the mutation status of other common AML co-mutation genes. The median follow-up time was 12.93 (0.37–53.53) months. Table 2 and Additional file 2A revealed that older patients (age ≥ 60 years) had a shorter OS (*P * = 0.034). Higher WBC counts (≥ 50 × 10^9^/L) were associated with a shorter OS (*P* = 0.035, Additional file 2C) and EFS (*P* = 0.006, Additional file 2D). Cases who accepted allo-HSCT had a longer OS (*P* = 0.024, Additional file 2E) and a better EFS (*P* = 0.013, Additional file 2F). The adverse risk group had a lower OS (*P* = 0.005) and EFS (*P* = 0.004). *AML1-ETO* coexistence was related to a prolonged OS (*P* = 0.010, Additional file 3A) and EFS (*P* = 0.013, Additional file 3B). *FLT3-ITD* co-mutation was related to a shorter OS (*P* < 0.001, Additional file 3C) and EFS (*P* < 0.001, Additional file 3D). However, neither the *ASXL1* mutation sites nor the *ASXL1* VAF had impacts on EFS or OS.

**Table 2:** Comparison of EFS and OS between different clinical and molecular characteristic groups in *ASXL1*+ AML

Variables	OS		EFS	
	*χ*^2^	*P*-value	*χ*^2^	*P*-value
Sex (female vs. male)	0.69	0.406	0.719	0.395
Age (≥ 60 vs. < 60 years)	4.513	0.034	2.96	0.085
*ASXL1* type (n [%])				
G652S	0.911	0.34	1.528	0.216
G642fs	1.243	0.265	1.737	0.188
H630fs	0.592	0.442	0.214	0.643
* ASXL1* VAF (≥ 49.17% vs. < 49.17%)	0.005	0.944	0.344	0.557
WBC counts (≥ 50 vs. < 50 × 10^9^/L)	4.471	0.035	7.564	0.006
HGB counts (≥ 110 vs. < 110 g/L)	0.131	0.717	0.085	0.77
PLT counts (≥ 100 vs. < 100 × 10^9^/L)	1.216	0.27	2.674	0.102
Bone marrow blasts (≥ 80% vs. < 80%)	0.611	0.434	0.364	0.546
Peripheral blasts (≥ 20% vs. < 20%)	1.242	0.537	1.939	0.379
Risk group (high-risk vs. low/inter)	7.719	0.005	8.231	0.004
Allo-HSCT (yes vs.no)	5.066	0.024	6.105	0.013
* AML1-ETO* (positive vs. negative)	6.583	0.01	6.229	0.013
* CBFβ-MYH11* (positive vs. negative)	0	0.993	0.018	0.894
* TET2* (mutated vs. wild type)	0.738	0.39	1.206	0.272
* FLT3-ITD* (positive vs. negative)	14.081	0	11.395	0.001
* U2AF1* (mutated vs. wild type)	3.056	0.08	2.273	0.132
* CEBPA* (mutated vs. wild type)	0.954	0.329	0.476	0.49
* NRAS* (mutated vs. wild type)	1.338	0.247	0.986	0.321
* DNMT3A* (mutated vs. wild type)	1.824	0.177	1.205	0.272
* IDH2* (mutated vs. wild type)	3.21	0.073	2.199	0.138
* RUNX1* (mutated vs. wild type)	0.608	0.436	0.391	0.532
* KIT* (mutated vs. wild type)	1.557	0.212	1.949	0.163
* SRSF2*(mutated vs. wild type)	0.323	0.570	0.537	0.464

*VAF* variant allele frequency, *WBC* white blood cell, *HGB* hemoglobin, *PLT* platelet, *allo-HSCT* allogenic hematopoietic stem cell transplantation

The factors with *P *< 0.05 in univariate analyses were included in the multivariate analysis. *FLT3-ITD* co-mutation had an independent predictive impact on poor OS (Table 3). Allo-HSCT was an independent protective factor for the OS and EFS of *ASXL1+* AML patients (Table 3).

**Table 3:** Multivariate analysis for OS and EFS in *ASXL1*+ AML

Variables	OS		EFS	
	HR (95%CI)	*P*-value	HR (95%CI)	*P*-value
Age ≥ 60 years	1.382 (0.720–2.653)	0.331		
Risk group	1.734 (0.655–4.585)	0.268	2.219 (0.847–5.813)	0.105
Allo-HSCT	0.204 (0.061–0.085)	0.01	0.184 (0.056–0.605)	0.005
WBC counts (≥ 50 × 10^9^/L)	1.194 (0.566–2.517)	0.641	1.826 (0.862–3.867)	0.116
*FLT3-ITD*	2.894 (1.260–6.647)	0.012	1.848 (0.810–4.215)	0.144
*AML1-ETO*	0.611 (0.176–2.123)	0.438	0.760 (0.231–2.507)	0.653

*OS* overall survival, *EFS* event-free survival, *HR* hazard ratio, *CI* confidence interval, *allo-HSCT* allogenic hematopoietic stem cell transplantation, *WBC* white blood cell

Then, we assessed the prognosis effect of the aforementioned factors in the adverse-risk group. The survival study revealed that decreased HGB levels (< 110 g/L), *FLT3-ITD* mutations, and *RUNX1* mutations had a negative influence on the OS of *ASXL1*+ AML patients (*P *= 0.045, *P *= 0.047, and *P *= 0.027, respectively; Additional file 3E, F). These variables had no impact on EFS. Allo-HSCT recipients had a longer OS and EFS (*P *= 0.024 and *P *= 0.013, respectively). HGB levels < 110 g/L and the *FLT3-ITD* mutations were found to have an independent predictive influence on poor OS in the multivariate analysis.

### Increased number of risk factors may shorten the OS and EFS of *ASXL1*+ AML patients

**Fig. 2:**
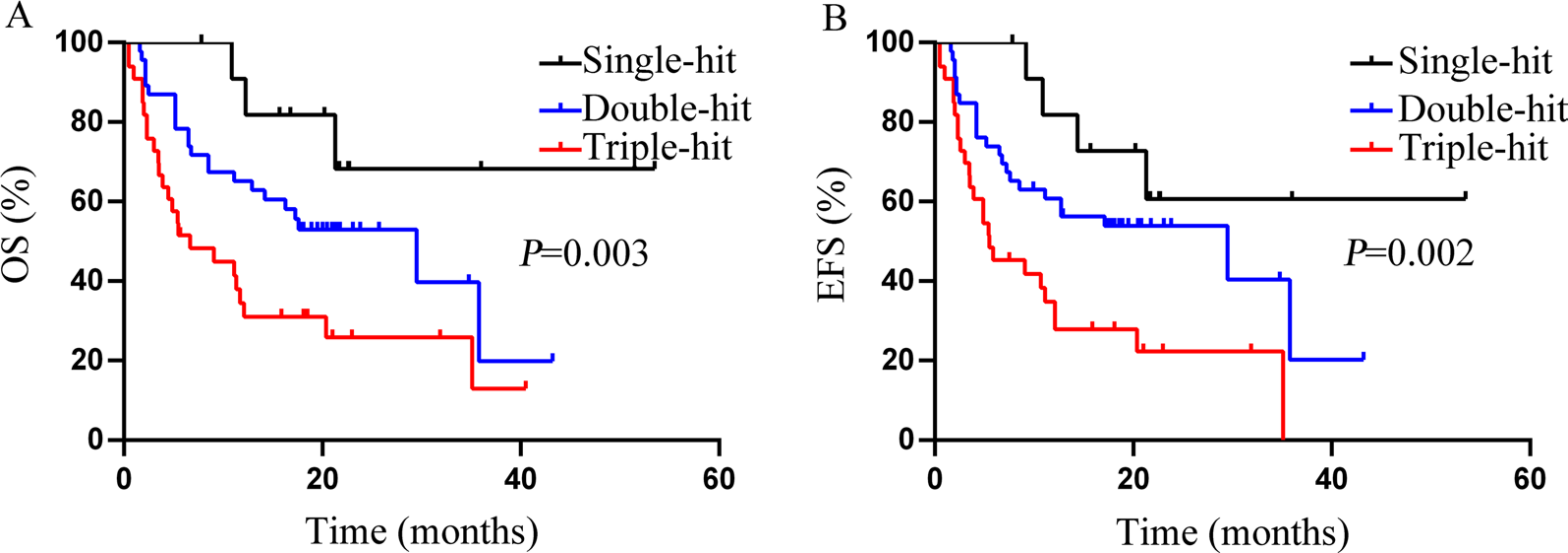
Comparison of OS (**A**) and EFS (**B**) in single-hit, double-hit and triple-hit *ASXL1+* AML. The factors that had adverse impact on OS and EFS are defined as high risk factors, including age ≥ 60 years, WBC count ≥ 50 × 10^9^/L, *FLT3-ITD* mutations, *RUNX1* mutations, and absence of *AML1-ETO* fusion gene. *ASXL1* mutations without any risk factor were classified as single-hit *ASXL1*+ AML. *ASXL1* mutations together with any of the risk factors was referred to as double-hit *ASXL1*+ AML. *ASXL1* mutations along with any two or more of the risk factors were designated as triple-hit *ASXL1*+ AML

The aforementioned factors that had adverse impact on OS and EFS are defined as high risk factors, including age ≥ 60 years, WBC count ≥ 50 × 10^9^/L, *FLT3-ITD* mutations, *RUNX1* mutations, and the absence of *AML1-ETO* fusion gene. *ASXL1* mutations without any risk factor were referred to as single-hit *ASXL1*+ AML. *ASXL1* mutations with one risk factor was referred to as double-hit *ASXL1*+ AML. *ASXL1* mutations with two or more risk factors were referred to as triple-hit *ASXL1*+ AML. The combination of these risk factors had a negative influence on the prognosis of *ASXL1*+ AML (Fig. 2). The median OS was not attained in single-hit *ASXL1*+ AML, 29.53 months in double-hit *ASXL1*+ AML, and 6.67 months in triple-hit *ASXL1*+ AML (*P *= 0.003, Fig. 2A). The median EFS in single-hit *ASXL1*+ AML was not attained in single-hit *ASXL1*+ AML, 29.53 months in double-hit *ASXL1*+ AML, and 5.47 months in triple-hit *ASXL1*+ AML (*P *= 0.003, Fig. 2B).

### Allo-HSCT improved the survival of double/triple-hit *ASXL1*+ AML patients

**Fig. 3:**
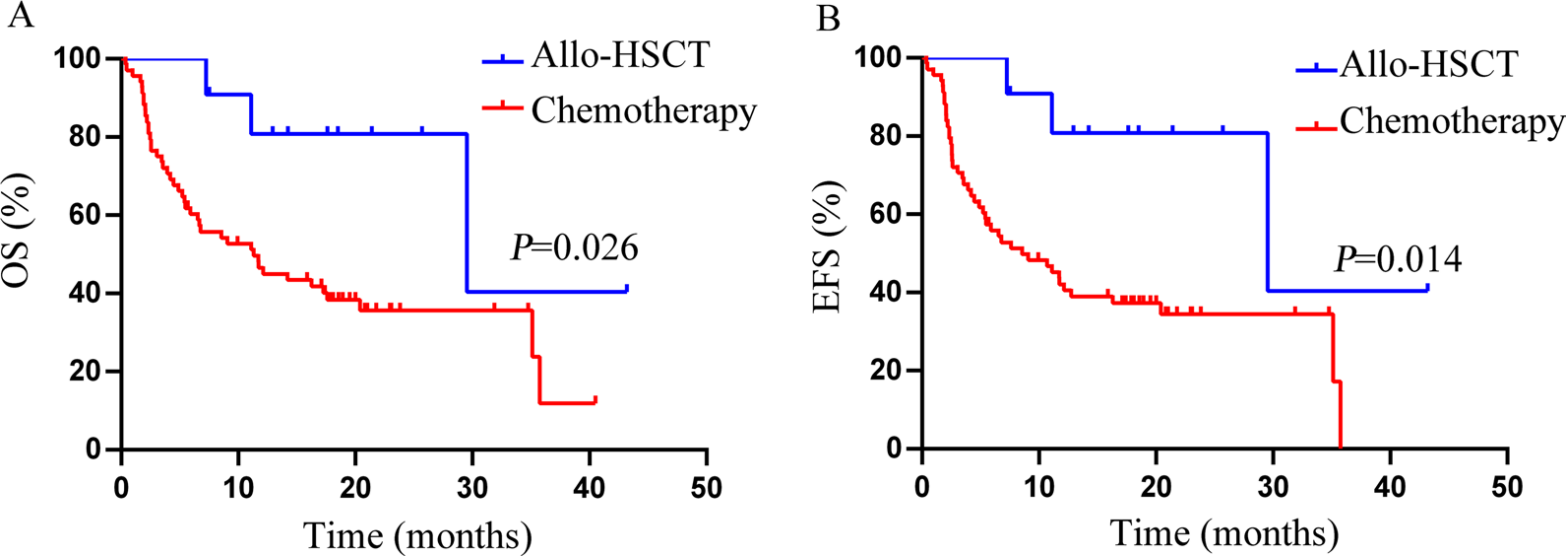
Transplantation can improve OS (**A)** and EFS (**B**) of *ASXL1*+ AML patients. The factors that had adverse impact on OS and EFS are defined as high risk factors, including age ≥ 60 years, WBC count ≥ 50 × 10^9^/L, *FLT3-ITD* mutations, *RUNX1* mutations, and absence of *AML1-ETO* fusion gene. *ASXL1* mutations without any risk factor were classified as single-hit *ASXL1*+ AML. *ASXL1* mutations together with any of the risk factors was referred to as double-hit *ASXL1*+ AML. *ASXL1* mutations along with any two or more of the risk factors were designated as triple-hit *ASXL1*+ AML

In our study, 12 patients received allo-HSCT as the consolidation management. Eleven of them carried one or more risk factors in addition to *ASXL1* mutations. As shown in Fig. 3, allo-HSCT significantly improved the OS (median 29.53 months vs. 11.33 months, *P *= 0.008, Fig. 3A) and EFS (median 29.53 months vs. 8.53 months, *P *= 0.007, Fig. 3B) in double or triple-hit *ASXL1*+ AML patients.

## Discussion

Previous researches found that *ASXL1* mutations were recurrent in 5–20% of AML patients[24–29]. These mutations are heterozygous and result in *ASXL1* mutants with a C-terminal truncation[21]. This *ASXL1* mutation pattern is characterized by dominant-negative or gain-of-function mutations [30]. *ASXL1* gain-of-function mutations have been linked to poor outcomes in AML patients [27, 29, 31, 32]. Based on the adverse outcome of *ASXL1*+ AML patients, *ASXL1* mutations were recognized as a stratification criterion for AML in the 2017 ELN guideline [4]. However, given the prevalence and adverse outcome of *ASXL1* mutations in AML, it is critical to identify the molecular landscape of *ASXL1*+ AML patients for establishing precise risk stratification in this subgroup of AML. This study investigated several key issues related to *ASXL1*+ AML, and discovered that the addition of other risk factors to *ASXL1* mutations worsens the adverse outcome of *ASXL1*+ AML patients.

The majority of *ASXL1* mutations in our study were found on codon 12, which is consistent with earlier reports [25, 33]. The most prevalent mutation was a guanine duplication (c.1934dupG) that results in a frameshift (p.Gly646TrpfsX12) [29, 34, 35]. These *ASXL1* mutations in AML patients are regarded as gain-of-function with a negative prognosis [30]. In our study, the distribution of mutation sites was quite diverse, with G652S, G642fs, and H630fs having the highest occurrence. This diversity of nucleotide mutations might be attributed to differences in the selected population and races. There was no statistical difference in OS and EFS across different mutated nucleotides, consistent with a recent study [35]. Furthermore, we found that *ASXL1* VAF did not correlate with survival, consistent with Richardson and colleagues’ findings that VAFs of *ASXL1* mutations were not significantly associated with OS [14]. Moreover, the current chemotherapy regimen and allo-HSCT may partly overcome the poor prognosis of high VAF and different mutation sites. The functional relevancies of *ASXL1* mutation in different nucleotides or frequencies are needed to further study.

Age ≥ 60 years and WBC counts ≥ 50 × 10^9^/L are typically linked with unfavorable risk and poor outcome in AML patients [4]. In our cohort of *ASXL1*+ AML, age ≥ 60 years also had a detrimental influence on OS. WBC account ≥ 50 × 10^9^/L had a negative impact on OS and EFS. Allo-HSCT, which was formerly thought to be the cure for AML, showed a survival advantage, particularly in individuals with double or triple-hit *ASXL1*+ AML.

Most primary MDS patients with *ASXL1* mutations (85%) have concurrent mutations of genes at the time of diagnosis [36]. The mutational profiles of *ASXL1*+ AML are complicated and multiple molecular interactions may exist. We observed that 86.46% of *ASXL1*+AML patients had additional gene mutations. Detailed investigation in the roles of co-occurred mutations is necessary for *ASXL1*+ AML patients. A previous study showed that *RUNX1* mutation promotes leukemogenesis of myeloid malignancies in *ASXL1*+ leukemia [37] and is associated with adverse prognoses of patients with de novo AML [38]. In our study, *RUNX1* did not have effects on OS and EFS in the overall prognostic analysis, but it was associated with shorter OS in high-risk *ASXL1*+ AML patients. This suggests that *RUNX1* mutation does not have prognostic significance in *ASXL1*+ AML and may be involved in the leukemogenesis of this subtype of AML. In addition, *FLT3-ITD* mutation is recognized as a poor prognostic factor that is associated with short OS, EFS and DFS [38–40]. The *ASXL1*, *FLT3-ITD*, and *RUNX1* mutations have been identified as major risk factors in AML patients by the ELN guidelines [4]. In this study, *FLT3-ITD* mutations were also associated with a shorter OS and EFS in *ASXL1*+ AML patients and correlated with a shorter survival time in high-risk *ASXL1*+ AML patients. This finding suggests that *FLT3-ITD* mutations exacerbate the poor prognosis of *ASXL1*+ AML. The *AML1-ETO* fusion gene results from the chromosomal translocation t(8;21), and is usually related to good response to induction therapy, as well as high complete remission rates in AML patients [41]. Our findings showed that the *AML1-ETO* fusion gene was similarly associated with a prolonged OS and EFS in *ASXL1*+ AML patients. These data demonstrate that the complex molecular interactions may affect the prognosis of *ASXL1*+ AML patients. Our study further identified the factors associated with prognostic heterogeneity in *ASXL1*+ AML patients. The application of multiple-hit theory may improve the prognostic stratification schemes, making the prognosis in *ASXL1*+ AML more precise. Future studies can also formulate a potential scoring system with these prognostic factors after validated on large cohort of *ASXL1*+ AML cases. As a result, clinicians can develop individualized precision treatment options for each patient.

Currently, clinical diagnoses and risk assessments for AML are mostly based on cytogenetic and genomic changes [4]. The prognosis for AML patients varies substantially, particularly for those with normal karyotype [31]. With the application of NGS in the clinical practice, we can better understand the complex roles and prognostic impacts of molecular mutations of genes in AML. According to the multiple-hit theory of genetic alterations in lymphoma and multiple myeloma, we further analyzed the additional risk factors for the survival of *ASXL1*+ AML patients. The results showed that the more risk factors, the shorter the OS and EFS for *ASXL1*+ AML patients. The application of allo-HSCT significantly improved the prognosis of *ASXL1*+ AML patients [35]. This was also applicable to the double-hit/triple-hit patients defined in our study, further confirming the importance of allo-HSCT in the treatment of AML patients.

Our research had several limitations. First, our study was retrospective and prone to selection biases. Second, owing to technical limitations, certain gene mutations may go undetected. Prognostic implications of some gene mutations may be overlooked. Third, the small sample sizes of several subgroups resulted in relatively low statistical power. Because of these constraints, our findings require confirmation in a larger and prospective population.

## Conclusions

This study provides new insights into the mutational spectrum and prognostic factors of *ASXL1*+ AML patients. The results demonstrate that increasing risk factors are associated with adversary prognosis of *ASXL1*+ AML patients. Our research further emphasizes the necessity of having the precise risk stratification for *ASXL1*+ AML patients.

## Supplementary Information

**Additional file 1.** *ASXL1* mutations at codon 12 of 91 de novo AML patients. Distribution and frequencies are given for *ASXL1 *mutations at codon 12. The boxes in one column represent single patient case. Mutations were color coded by mutation type. The histogram on the right showed the frequency distribution of all aberrations.

**Additional file 2.** Comparison of OS and EFS between different clinical characteristic groups in *ASXL1+ *AML. OS and EFS were compared in (A-B) patients older than 60 years and patients younger than 60 years; (C-D) patients with WBC≥50 × 10^9^/L vs. <50 × 10^9^/L; (E-F) patients who accepted　allo-HSCT or not.

**Additional file 3.** Comparison of OS and EFS between different　clinical characteristic groups in *ASXL1+ *AML. OS and EFS were compared in (A-B) patients　with *AML1-ETO* fusion gene or not; (C-D) patients with *FLT3-ITD　*mutations or not; (E-F) adverse-risk patients with *RUNX1* mutations or　not.

## Data Availability

The data that support the findings of our research are available from The First Affiliated Hospital of Zhengzhou University, but restrictions apply to the availability of these data, which were used under license for the current study, and so are not publicly available.

## Abbreviations

ASXL1: Additional sex combs-like 1
Asx: Additional sex combs
AML: Acute myeloid leukemia
APL: Acute promyelocytic leukemia
MDS: Myelodysplastic syndromes
allo-HSCT: Allogenic hematopoietic stem cell transplantation
HSCT: Hematopoietic stem cell transplantation
RAR: Retinoic acid receptor
NGS: Next-generation sequencing
EDTA: Ethylene diamine tetraacetic acid
VAF: Variant allele frequency
WBC: White blood cell
HGB: Hemoglobin
PLT: Platelet
OS: Overall survival
EFS: Event-free survival
