# Supplementary figures and images for: Risk factors affect accurate prognosis in ASXL1-mutated acute myeloid leukemia
Source: Cancer Cell Int. 2021 Oct 9;21:526. doi: 10.1186/s12935-021-02233-y (PMC8502294; doi:10.1186/s12935-021-02233-y)

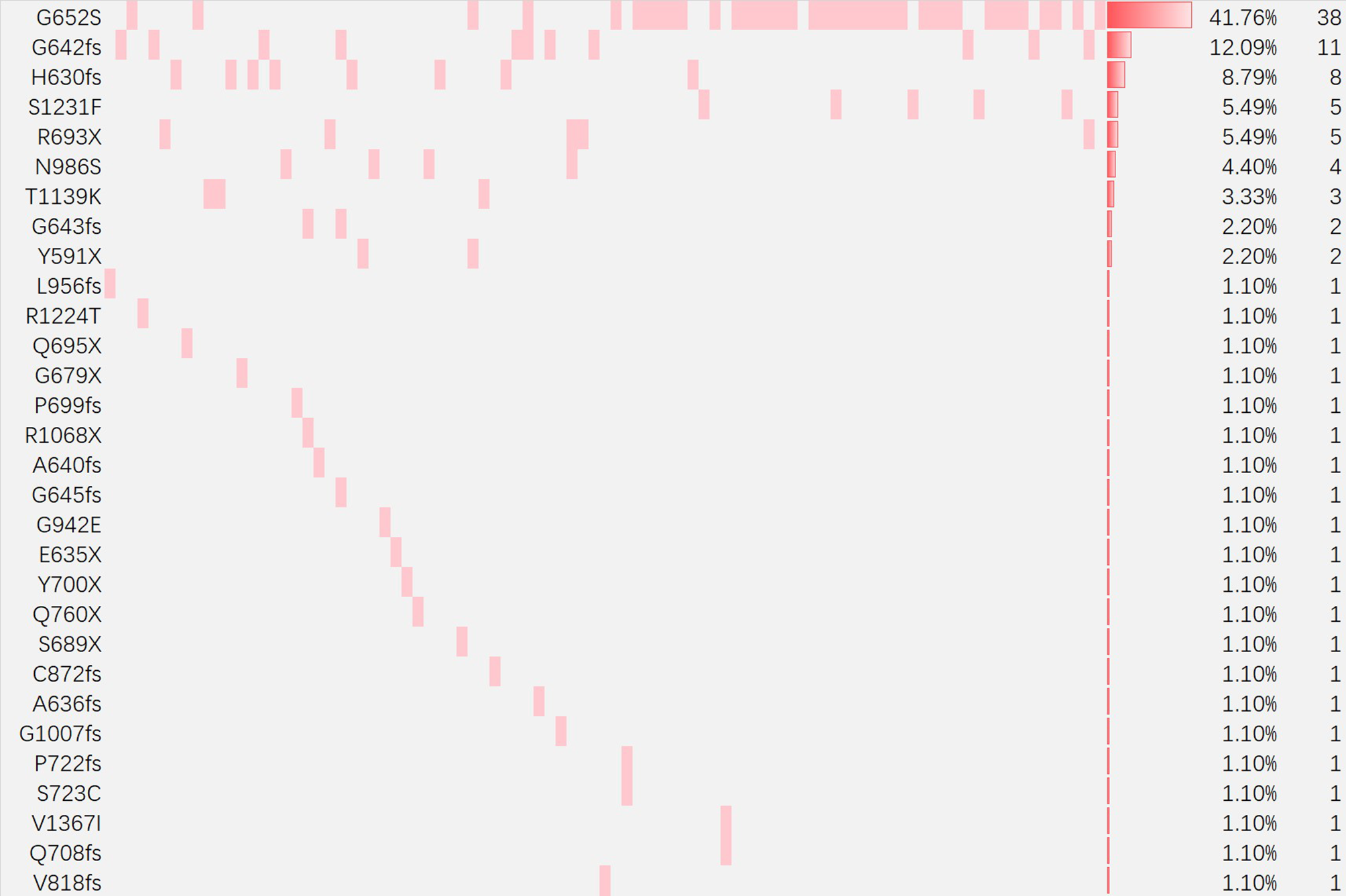

Supplement: Supplementary file 1 — Additional file 1. ASXL1 mutations at codon 12 of 91 de novo AML patients. Distribution and frequencies are given for ASXL1 mutations at codon 12. The boxes in one column represent single patient case. Mutations were color coded by mutation type. The histogram on the right showed the frequency distribution of all aberrations. [file 12935_2021_2233_MOESM1_ESM.tif]
